# DETERMINANTS OF RECOVERY IN THE US MEDICARE MINIMUM DATA SET: HANDLING INFORMATIVE OBSERVATION TIMES

**DOI:** 10.1093/geroni/igae098.2084

**Published:** 2024-12-31

**Authors:** Michelle Shardell, Chixiang Chen, Jason Falvey

**Affiliations:** University of Maryland School of Medicine, Baltimore, Maryland, United States; University of Maryland School of Medicine, Baltimore, Maryland, United States; University of Maryland School of Medicine, Baltimore, Maryland, United States

## Abstract

Patients with Alzheimer’s Disease (AD) who experience a hip fracture are heterogeneous and understudied; thus, factors that influence these patients’ physical recovery are unknown. Moreover, identifying these factors can help inform personalized care for this population. To fill this gap, we use the US Minimum Data Set (MDS), a federally mandated standardized clinical assessment tool administered by the United States Centers for Medicare and Medicaid Services to facilitate care management for residents in Medicare and Medicaid certified nursing homes. Longitudinal assessments in these real-world data are irregular, and their timing is likely informative, which leads to bias if ignored. Therefore, we introduce and apply a novel joint model for longitudinal outcomes and observation times that includes a shared random effect. Data include 9,545 older adults (76.8% female, 55.7% aged 86+ years, 21.8% Medicaid eligible) in the MDS living with AD who were discharged from the hospital after hip fracture to Medicare and Medicaid certified nursing homes between 2017 and 2022. Barthel Index, a measure of physical function in activities of daily living, was the outcome (range: 0 to 90, higher scores indicate better function). Participants underwent 35,794 assessments over 100 days. Medicaid eligibility and lower cognition measured using the Brief Interview for Mental Status were more strongly associated with worse physical function in the proposed method than in a competing method (p<.05). Findings highlight 1) potential roles of socioeconomic status and heterogeneous cognition in AD in hip fracture recovery, and 2) importance of addressing informative observation times in the MDS.

